# A free forum for neurosurgery and neuroscience

**DOI:** 10.4103/2152-7806.66619

**Published:** 2010-07-21

**Authors:** Pieter L. Kubben

**Affiliations:** Department of Neurosurgery, Maastricht University Medical Center, The Netherlands

In the June editorial on Neurosurgery 2.0, you have read about the so-called Web 2.0 technologies: wikis, online video, podcasts, RSS feeds, blogs, and social networks like Twitter and Facebook. At the same time, we implemented a new feature for the website that we consider to be of added value: a forum.

## WHAT IS A FORUM?

An internet forum, or message board, is an online discussion site.[2] It originated from the standard bulletin boards (or cork pinboards), where people could stick short notes to announce, sell or request something. The digital variant has more to offer:

1. Web based, so universally accessible
2. Users can reply to messages and subscribe to other replies
3. A “search” function allows to look for all topics on a specific subject

Although a forum should be moderated to make sure topics are in the right category and questions are addressed properly, forum users remain responsible for how they use the content. For that reason, it is required to create an account. Forum accounts for “Surgical Neurology International” are free but are required for various reasons. On the positive side, we want to reward people who make significant forum contributions to help other colleagues. On the defensive side, we want to protect you against spam messages. The latter requires registration with a valid e-mail address and solving a so-called CAPTCHA.

## WHAT IS A CAPTCHA?

A CAPTCHA (or Captcha) is a type of challenge-response test used in computing to ensure that the response is not generated by a computer.[1] The contrived acronym CAPTCHA stands for “Completely Automated Public Turing test to tell Computers and Humans Apart”, and is, as the description reveals, a variant of the Turing test. The latter is a proposal for a test of a machine’s ability to demonstrate intelligence by comparing human (written) language response with a machine’s language response. The machine is said to pass the test if a blinded observer cannot determine which response is generated by the computer (which has not happened yet).[4]

A CAPTCHA works by showing the user distorted letters and/ or numbers and asking to type them on the computer keyboard. The greater the distortion, the more difficult it becomes for automated spam software (spam bots) to decipher the characters that are used. Humans are much better capable of solving this question, but of course there is a limit. A balance has to be maintained between “as difficult as possible” for spam bots and “as easy as possible” for humans.

For “Surgical Neurology International”, we chose to use reCAPTCHA.[3] This is a free CAPTCHA service that helps to digitize books, newspapers and old-time radio shows. reCAPTCHA improves the process of digitizing books by sending words that cannot be read by computers to the Web in the form of CAPTCHAs for humans to decipher. Each new word that cannot be read correctly by regular scanning software is given to a user in conjunction with another word for which the answer is already known. The user is then asked to read both words. If they solve the one for which the answer is known, the system assumes their answer is correct for the new one. The system then gives the new image to a number of other people to determine, with higher confidence, whether the original answer was correct.[3] So by registering for the forum of “Surgical Neurology International”, you contribute to saving our cultural heritage.

## FORUM CATEGORIES

The forum contains three categories: Clinical, News and Worldwide Market. In the Clinical category, you can share difficult cases and operative techniques. In the News category, there are headings for individual achievements, updates from neurosurgical or neuroscientific societies, and announcements for upcoming meetings. In the Worldwide Market category, there are headings for requesting or offering fellowships, volunteers, and equipment. In all these categories, you can post topics and you can react to topics that other people post.

## DIFFERENCE BETWEEN AN ONLINE FORUM AND A BLOG

If an online forum is an online discussion site, then how is it different from a blog? Both in the blog (the “Posts” section on our website) and in the forum, you can add replies to topics; but only in the forum, you can directly add topics without any editorial approval. We require you to register for the forum for the reasons explained in this editorial. These are less applicable to the blog for a variety of reasons: different nature of the topics, and other ways to protect against spam.

Both the blog and the forum offer RSS feeds to easily keep you up-to-date. Also, both the blog and the forum are connected to our three most important social networks: Twitter, Facebook and LinkedIn. If you use any of these, you will automatically be notified of updates in the blog and the forum. We are working on the same feature for journal articles, but at the moment there are some technical issues that require us to update Twitter and Facebook manually for this goal.

## NOW WHAT?

You can visit the website of “Surgical Neurology International” at http://surgicalneurologyint.com, where you will find the heading “Forum” in the menu. This offers access to the forum index page, as well as all subcategories. You can read all topics and replies without registration. If you wish to write a topic or post your reply to another topic, you’ll find the “Register” link on top of the screen. Welcome to the community!
